# Asynchrony enhances uncanniness in human, android, and virtual dynamic facial expressions

**DOI:** 10.1186/s13104-023-06648-w

**Published:** 2023-12-11

**Authors:** Alexander Diel, Wataru Sato, Chun-Ting Hsu, Takashi Minato

**Affiliations:** 1 Cardiff University School of Psychology, Cardiff, UK; 2 RIKEN Institute, Kyoto, Japan; 3 Clinic for Psychosomatic Medicine and Psychotherapy, LVR University Hospital Essen, University of Duisburg-Essen, 45147 Essen, Germany; 4 Center for Translational Neuro- and Behavioral Sciences (C-TNBS), University of Duisburg- Essen, 45147 Essen, Germany

**Keywords:** Asynchrony, Configural processing, Dynamic face emotion expression, Inversion effect, Uncanny valley

## Abstract

**Objective:**

Uncanniness plays a vital role in interactions with humans and artificial agents. Previous studies have shown that uncanniness is caused by a higher sensitivity to deviation or atypicality in specialized categories, such as faces or facial expressions, marked by configural processing. We hypothesized that asynchrony, understood as a temporal deviation in facial expression, could cause uncanniness in the facial expression. We also hypothesized that the effect of asynchrony could be disrupted through inversion.

**Results:**

Sixty-four participants rated the uncanniness of synchronous or asynchronous dynamic face emotion expressions of human, android, or computer-generated (CG) actors, presented either upright or inverted. Asynchrony vs. synchrony expressions increased uncanniness for all upright expressions except for CG angry expressions. Inverted compared with upright presentations produced less evident asynchrony effects for human angry and android happy expressions. These results suggest that asynchrony can cause dynamic expressions to appear uncanny, which is related to configural processing but different across agents.

## Introduction

Artificial humanoids approximating realistic appearances can appear eerie, strange, or uncanny [1]. While humanlike robots can take on various service roles [2–5], their uncanniness can inhibit trust in human-machine interaction [6]. Investigating the causes of uncanniness in artificial entities is vital to the design of acceptable artificial humanoids.

Facial deviations can cause uncanniness [7–13]. Humans are specialized for faces which sensitizes the detection of subtle uncanny anomalies [8]. Specialization in faces is marked by a sensitivity to configural information specifically for upright faces [14–16]. Global inversion disrupts this configural processing, reducing the sensitivity to facial structure [17, 18]. As inversion decreases the accuracy of facial aesthetics ratings [19–21], face aesthetics may also rely on configural information. Accordingly, facial deviations are uncannier in upright compared to inverted faces [8].

Emotional expressions can be defined as the configuration of face muscle motion across time [22, 23]. Dynamic information (i.e., the sequence of face muscle motion) specifically binds the muscle motion into a configural whole [24]. Asynchronous face motion can thus be considered a configural deviation. Yet the effect of asynchrony specifically on uncanniness has not yet been investigated. We hypothesized that, if deviation causes uncanniness in general, asynchronous motion should appear uncanny as well. Moreover, the asynchrony effects of uncanniness would be reduced through inversion.

Finally, specialization degrees differ between types of faces or agent: The inversion effect is smaller in less realistic faces [25–31]. As specialized processing is thought to sensitize stimuli to deviation, leading to uncanniness, a lower level of specialized processing for less realistic faces may explain why such faces are less affected by deviations [8, 11, 12]. Inversion effects on the uncanniness of asynchronous motion should thus be more strongly pronounced in more realistic faces, specifically faces of embodied entities like human and android stimuli: Inversion effects are present for highly humanlike robot faces [29], but are decreased for CG faces [25–27]. A higher specialization for android faces may sensitize the detection of facial anomalies, increasing the likelihood that the entity appears eerie or creepy.

Thus, the following three hypotheses are tested:

1. Asynchronous motion in facial expression increases uncanniness (*asynchrony effect*).
2. Inversion reduces the effect of asynchrony on uncanniness (*uncanniness inversion effect*).
3. The uncanniness inversion effect is present in humans and androids but not in CG expressions (*actor effect*).

## Methods

### Participants

Power analysis using Pangea [32] was conducted using a 3 (actor) times 2 (emotion) times 2 (orientation) times 3 (asynchrony) design, a (medium) effect size of *d* = 0.5, α = 0.05, and 1 - β = 0.80. The analysis concluded n = 64 to be sufficient for the analysis. Sixty-four Japanese volunteers were recruited for this study (31 = female, 31 = male, two not specified, *M*_age_ = 30.65, *SD*_age_ = 3.88 years) using CrowdWorks (Tokyo, Japan).

### Materials

Nikola, an android with 35 pneumatic actuators to simulate facial muscles for emotion expression, was used [33]. Nikola’s actuators allow for a temporal resolution in the millisecond range, and are hence used to recreate asynchronous motion of face emotion expressions. Angry and happy human videos were taken from the AIST database [34]. Angry and happy CG videos were designed via FACSGen [35, 36].

A set of 36 videos were created, consisting of 3 actors (human, android, CG), 3 asynchrony levels (synchronous, 250ms delay, 500ms delay), 2 orientations (upright, inverted), and 2 emotions (angry, happy). All videos were cut at the actor’s neck, head, and ears, and actor noses were at the same height, and had a white background. Video length was cut to be 1.25 s and showed the onset of the emotion expressions viewed from the front.

**Fig. 1:**
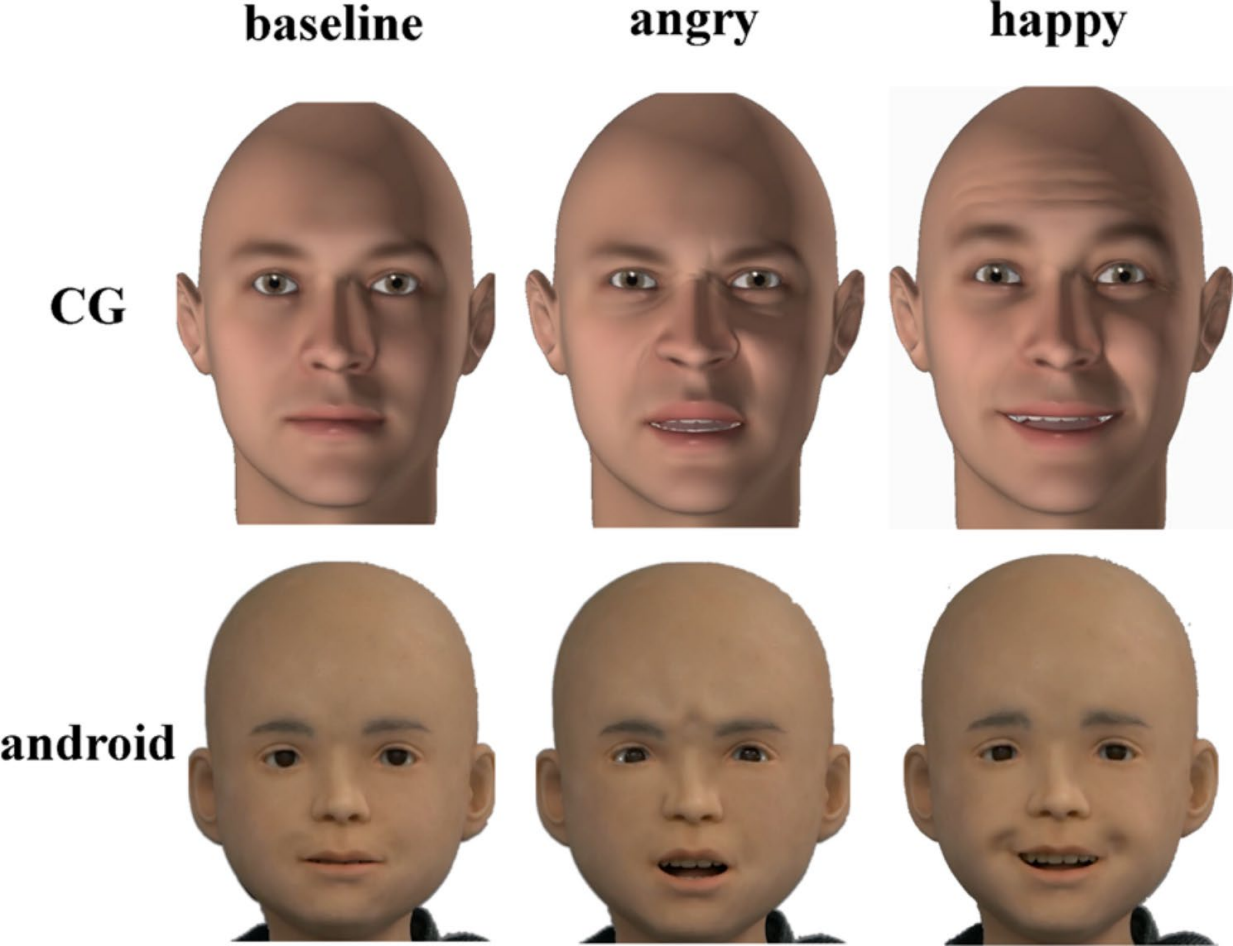
Android and CG stimuli divided by emotion and asynchrony level. Note: Human stimuli are not shown as the AIST database prohibits the distribution of their stimulus material

Asynchronies were manipulated by delaying a face’ upper right and left half motions. For the 250ms delay condition, the upper right half motion was delayed 250ms and the upper left 500ms; for the 500ms delay condition, the upper right half motion was delayed 500ms and the upper left 1000ms. Android and CG stimuli are depicted in Fig. 1.

### Procedure

An online-based design was used. Participants were linked to the experiment page after providing informed consent. All videos were shown in a randomized order, and participants were asked to rate videos on the three scales *uncanny*, *strange*, and *humanlike*, which are effective measures of the uncanny valley effect [37]. Scales ranged from 0 to 100 and could rewatch the videos an unlimited amount while simultaneously rating each video.

### Statistical analysis

A within-participant ANOVA with actor type, orientation, distortion, and emotion was conducted to test for interaction effects on uncanniness. Post hoc tests with Bonferroni-adjusted p-values were then conducted to test group differences relevant to the hypotheses. The analysis was conducted using R (version 4.1.2).

## Main text

### Differences between conditions

A within-participant ANOVA with actor type, orientation, emotion, and distortion as factors revealed that the highest 4-way interaction was significant (*F*(2,44) = 4.16, *p* = .022, *η*^2^_p_ = 0.16), as well as the existence of significant interactions between type and distortion (*F*(2,44) = 5.09, *p* = .01 *η*^2^_p_ = 0.19), orientation and emotion (*F*(2,44) = 19.28, *p* < .001, *η*^2^_p_ = 0.3), and type and emotion (*F*(2,44) = 3,46, *p* = .04, *η*^2^_p_ = 0.14).

Post-hoc Tukey tests were conducted to test for differences between distortion levels across orientations and actor types (Tables 1 and 2).

**Fig. 2:**
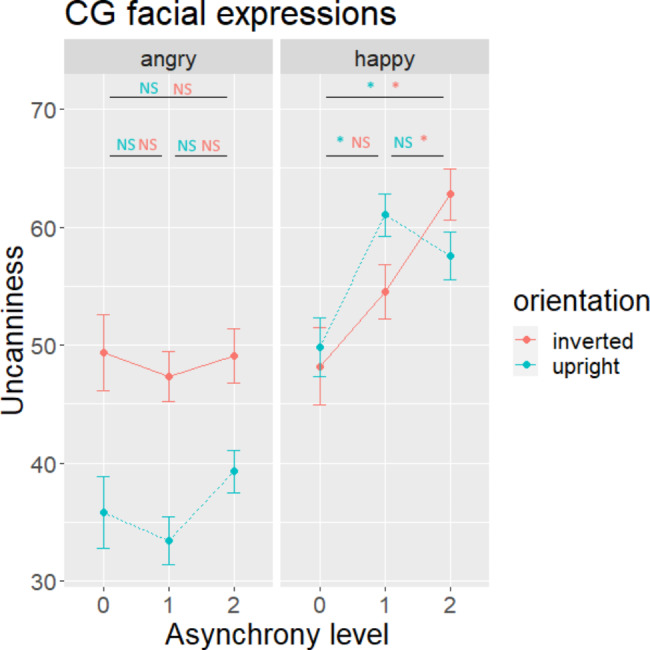
Mean uncanniness ratings for human expressions divided by emotion (angry, happy), distortion (asynchrony) level, and orientation. Note: Error bars indicate standard errors. Asterisks indicate significant differences while *NS* indicates non-significant differences. Blue (first) significant marks are for upright, and red (last) significant marks are for inverted conditions. For each emotion, differences were tested between distortion (asynchrony) levels 0 to 2 (upper line), 0 to 1 (lower left line), and 1 to 2 (lower right line), color-coded for orientation

For human expressions (Fig. 2), asynchronies increased uncanniness for upright-angry (levels 0 vs. 2), upright-happy and inverted-happy expressions (levels 0 vs. 1), but not for inverted-angry expressions.

**Fig. 3:**
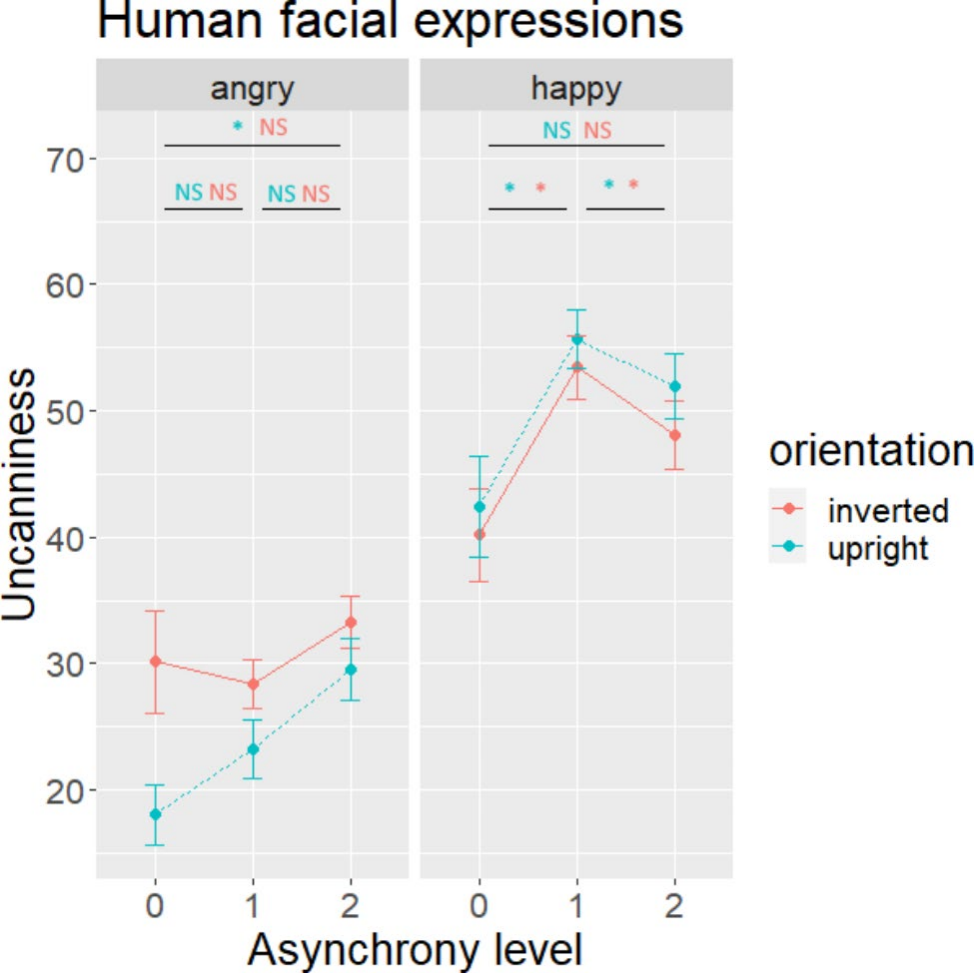
Mean uncanniness ratings for android expressions divided by emotion (angry, happy), distortion (asynchrony) level, and orientation. Note: Error bars indicate standard errors. Asterisks indicate significant differences while *NS* indicates non-significant differences. Blue (first) significant marks are for upright, and red (last) significant marks are for inverted conditions. For each emotion, differences were tested between distortion (asynchrony) levels 0 to 2 (upper line), 0 to 1 (lower left line), and 1 to 2 (lower right line), color-coded for orientation

For android expressions (Fig. 3), asynchronies increased uncanniness for upright-angry (0 vs. 2 levels), inverted-angry (0 vs. 1 and 0 vs. 2 levels), and upright-happy (0 vs. 1 and 0 vs. 2 levels) expressions, but not for inverted-happy expressions.

**Fig. 4:**
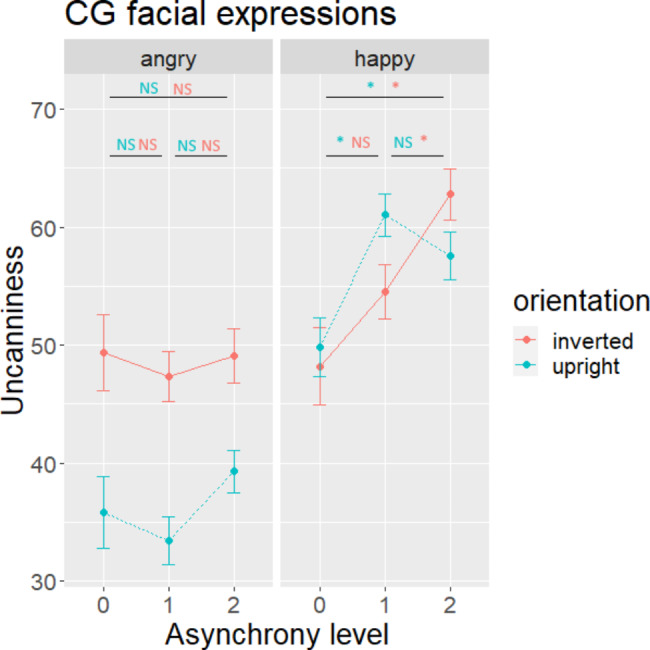
Mean uncanniness ratings for CG expressions divided by emotion (angry, happy), distortion (asynchrony) level, and orientation. Note: Error bars indicate standard errors. Asterisks indicate significant differences while *NS* indicates non-significant differences. Blue (first) significant marks are for upright, and red (last) significant marks are for inverted conditions. For each emotion, differences were tested between distortion (asynchrony) levels 0 to 2 (upper line), 0 to 1 (lower left line), and 1 to 2 (lower right line), color-coded for orientation

For CG expressions (Fig. 4), asynchronies increased uncanniness for upright-happy (0 vs. 1 levels) and inverted-happy (0 vs. 2 levels) expressions, but not for upright-and inverted-angry expressions.

**Table 1:** Test statistics of each post-hoc test of distortion (asynchrony) difference performed across orientation and actor type, for angry expressions

Emotion	Actor	Orientation	Distortion difference	t-value	*p*_adj_-value	Effect size (d)
angry	human	upright	0–1	*t*(1060) = -1.36	0.26	
			0–2	*t*(1060) = -2.09	0.055	
			1–2	*t*(1060) = -3.15	0.002*	0.63
		inverted	0–1	*t*(1060) = 0.35	1	
			0–2	*t*(1060) = -0.88	0.57	
			1–2	*t*(1060) = -1.46	0.22	
	android	upright	0–1	*t*(1060) = -2.26	1	
			0–2	*t*(1060) = -3.86	< 0.001*	0.78
			1–2	*t*(1060) = -4.54	< 0.001*	0.73
		inverted	0–1	*t*(1060) = -2.32	0.031*	0.25
			0–2	*t*(1060) = -3.64	0.003*	0.38
			1–2	*t*(1060) = -0.98	0.485	
	CG	upright	0–1	*t*(1060) = 0.51	1	
			0–2	*t*(1060) = -0.92	0.54	
			1–2	*t*(1060) = -1.77	0.116	
		inverted	0–1	*t*(1060) = 0.36	1	
			0–2	*t*(1060) = 0.04	1	
			1–2	*t*(1060) = 0.4	1	

**Table 2:** Test statistics of each post-hoc test of distortion (asynchrony) difference performed across orientation and actor type, for happy expressions

Emotion	Actor	Orientation	Distortion difference	t-value	*p*_adj_-value	Effect size (d)
happy	human	upright	0–1	*t*(1075) = -3.2	0.002*	0.64
			0–2	*t*(1075) = -2.31	0.032*	0.46
			1–2	*t*(1075) = -1.09	1	
		inverted	0–1	*t*(1075) = -3.93	< 0.001*	0.73
			0–2	*t*(1075) = -2.44	0.02*	0.46
			1–2	*t*(1075) = -1.46	1	
	android	upright	0–1	*t*(1075) = -4.24	< 0.001*	0.87
			0–2	*t*(1075) = -3.16	0.002*	0.67
			1–2	*t*(1075) = 1.23	1	
		inverted	0–1	*t*(1075) = -1.49	0.21	
			0–2	*t*(1075) = -1.17	0.367	
			1–2	*t*(1075) = 0.36	1	
	CG	upright	0–1	*t*(1075) = -2.75	0.009*	0.55
			0–2	*t*(1075) = -2.26	0.04*	0.45
			1–2	*t*(1075) = 0.6	1	
		inverted	0–1	*t*(1075) = -1.61	0.162	
			0–2	*t*(1075) = -3.74	< 0.001*	0.79
			1–2	*t*(1075) = -2.72	0.01*	0.46

## Discussion

The effects of asynchrony on uncanniness were investigated, and how actor type and orientation influence these effects. Asynchrony increased uncanniness ratings for facial expressions under several conditions. The effect, however, also differed across orientation and actor type conditions.

According to hypothesis 1, asynchrony as a manipulation of deviating dynamic facial expressions should increase uncanniness. An increase in uncanniness was found across all upright expressions except for CG angry expressions. Thus, hypothesis 1 was confirmed for android and human agents. Previous research found that configural processing is used to process dynamic facial expressions [22]. Dynamic facial expressions may be processed by binding the sequence of face AU motions into a configural pattern. Deviations from this pattern, for example unusual timings of the face’s AU motions in relation to the other units, may create an atypical expression, which is detected through the configural processing of the expression dynamic and thus negatively evaluated.

Hypothesis 2 stated that the effect of asynchrony on uncanniness would decrease for inverted faces compared with upright ones. Inverted presentations produced fewer evident asynchrony effects for human angry and android happy expressions than did upright presentations. Thus, hypothesis 2 was supported for angry human and happy android expressions. Consistently, inversion effects on emotion recognition have been found to vary among different emotions [38–40]. We speculate that the specific facial movements of specific stimulus models (e.g., mouth opening) may explain the differences in the inversion effect. Since the inversion effect is used as an indicator for configural processing, the results suggest that asynchrony in an actor’s facial expressions are detected using a configural processing style at least in some expressions.

Our third hypothesis predicted that the asynchrony or asynchrony × inversion effects on uncanniness would be more obvious in humans and androids than in CG. As described above, the asynchrony effects for upright faces were evident for both angry and happy expressions for human and android expressions, but not for CG. Furthermore, inversion effects showing different patterns between inverted and upright conditions were partially found for humans and android expressions but not for CG. Previous research on static faces showed that human faces increase the recruitment of face-specialized configural processing compared to CG faces [27]. Similarly, humans are more sensitive to deviations in more realistic faces [8]. A higher level of realism in an actor may increase the sensitivity to deviations due to increased configural processing. Taken together, our results support our hypothesis indicating that the uncanniness of android and human faces is at least partially processed configurally, which is not the case for CG faces.

## Limitations

Only one type of asymmetrical asynchrony and emotion manipulation was used. The effects of specific asynchronies may differ across expressed emotions and thus not be generalized across other emotions and other types of asynchronies. Since only one variant per emotion was used, effects may also be different for other patterns of angry and happy expressions. Furthermore, asynchronies followed the same pattern with the upper right motion preceding upper left motion. Only one type was used to not overburden participants with the number of stimuli. Nevertheless, it is unclear whether the same results would be observed for a mirrored pattern.

The experimental android Nikola’s design is based on a child. Because configural processing is more pronounced for faces of a similar age, adult participants may have shown a decreased level of configural processing for this android specifically.

Furthermore, shading differed between conditions due to differences in lighting effects. Specifically, only CG faces showed clear shading effects. Although we are not aware of potential shading effects on configural processing, confounding shading effects cannot be excluded for observed differences between actors.

The patterns of results observed are not consistent: Specifically, inversion effects on asynchrony and on uncanniness were found only for happy human and angry android faces. Therefore, it is unclear to what degree the role of configural processing on the uncanniness of asynchronies can be generalized.

## Data Availability

Data, analysis, and android and CG video stimuli are available at https://osf.io/9cmhp. Human stimuli are not available because the AIST database prohibits distribution of their material.
